# Kounis Syndrome Features in Special Populations

**DOI:** 10.3390/medsci14020218

**Published:** 2026-04-28

**Authors:** Alexandr Ceasovschih, Nicholas G. Kounis, Sura Markos, Malik Ejubovic, Maria Cherska, Fotios Barkas, Vladimir Ristovski, Alexandru Corlateanu, Pradeesh Sivapalan, Stanislav Kotlyarov, Victorita Sorodoc, Laurentiu Sorodoc

**Affiliations:** 1 Grigore T. Popa University of Medicine and Pharmacy, 700115 Iasi, Romania; 2 Department of Cardiology, University of Patras Medical School, 26221 Patras, Greece; 3 Internal Medicine Department, Division of Cardiology, Hawassa University, Hawassa 1560, Ethiopia; 4 Department of Internal Medicine, Cantonal Hospital Zenica, 72000 Zenica, Bosnia and Herzegovina; 5 Department of Pathophysiology, University of Zenica, 72000 Zenica, Bosnia and Herzegovina; 6 Cardiology and Diagnostics Department, State Institution “V.P. Komisarenko Institute of Endocrinology and Metabolism of the National Academy of Medical Sciences of Ukraine”, 01001 Kyiv, Ukraine; emariya83@gmail.com; 7 Department of Internal Medicine, Faculty of Medicine, School of Health Sciences, University of Ioannina, 45221 Ioannina, Greece; 8 Department for Invasive and Interventional Cardiology, City General Hospital “8th of September”, 1000 Skopje, North Macedonia; 9 Department of Respiratory Medicine and Allergology, State University of Medicine and Pharmacy “Nicolae Testemitanu”, 2004 Chisinau, Moldova; alexandru.corlateanu@usmf.md; 10 Department of Clinical Medicine, Faculty of Health and Medical Sciences, University of Copenhagen, DK-2200 Copenhagen, Denmark; 11 Copenhagen Respiratory Research, Department of Medicine, Copenhagen University Hospital—Herlev and Gentofte, DK-2900 Copenhagen, Denmark; 12 Department of Nursing, Ryazan State Medical University, Ryazan 390026, Russia

**Keywords:** Kounis syndrome, allergy, hypersensitivity, anaphylactic reactions, special populations

## Abstract

Kounis syndrome (KS) describes the occurrence of acute coronary syndromes precipitated by allergic, hypersensitivity, or anaphylactic reactions and represents a unique intersection between immunologic activation and cardiovascular disease. The epidemiology of KS is likely underestimated due to diagnostic overlap with other cardiac and allergic conditions and limited awareness across medical specialties. This narrative review focuses on the distinctive features of KS in special populations, emphasizing how patients’ age, comorbidities, immune status, and vascular substrate modify presentation, diagnosis, and outcomes. In elderly patients, polypharmacy, increased plaque vulnerability, and endothelial dysfunction favor Type II and III KS. Pediatric cases, although rare, are predominantly Type I and strongly associated with food allergies, insect stings, vaccines, and antibiotics, with under-recognition driven by diagnostic bias and ethical concerns surrounding invasive testing. Patients with coronary stents, cardiac devices, chronic kidney disease, and those receiving dialysis exhibit heightened susceptibility due to chronic inflammation, foreign-body hypersensitivity, and prothrombotic states. Pregnancy and the peripartum period represent a unique immuno-hemodynamic milieu in which Th2 immune shift, increased coronary vasoreactivity, and obstetric triggers can compromise both maternal and fetal perfusion. Additional risk modulation is observed in atopic individuals, asthmatics, patients with autoimmune, inflammatory, oncologic, psychiatric, and neurodevelopmental conditions, as well as in COVID-19 and post-infectious states. We propose a host-modified framework for KS that complements traditional classification by integrating immune phenotype and vascular substrate, enabling improved risk stratification and personalized preventive strategies.

## 1. Introduction

Cardiovascular (CV) manifestations associated with allergic, hypersensitivity, anaphylactic, and anaphylactoid reactions have been reported in the medical literature for more than seven decades [1,2,3]. Early observations described “morphologic cardiac reactions” and “acute carditis,” primarily linked to serum sickness and tetanus antitoxin. The first documented case of acute myocardial infarction associated with urticaria was reported in 1950 in a patient receiving penicillin therapy [3]. The concept of allergic angina progressing to allergic myocardial infarction due to coronary vasospasm and endothelial dysfunction was formally described in 1991 [4]. Subsequent studies demonstrated extensive mast cell activation at sites of coronary plaque erosion and rupture, implicating mast cell–derived proteases in plaque destabilization [2,5]. Allergic angina was later classified as a form of dynamic coronary obstruction [3]. These observations culminated in the description of Kounis syndrome (KS), encompassing allergic angina and myocardial infarction triggered by mast cell activation [6]. Recent evidence confirms elevated cardiac biomarkers during anaphylaxis, highlighting the coronary arteries as primary targets [5,7].

KS is increasingly recognized as an important but still underdiagnosed cause of acute coronary syndromes triggered by allergic, hypersensitivity, anaphylactic, or anaphylactoid reactions [2,4,8]. Kounis syndrome is now recognized as a unique form of acute vascular syndrome that impacts the cerebral, mesenteric, peripheral, and venous systems in addition to the coronary arteries [9]. Although initially considered rare, accumulating clinical and epidemiological evidence suggests that KS affects patients of all ages, ethnic backgrounds, and geographic regions [1,3,10]. The true incidence of KS is likely underestimated due to low clinical awareness, frequent misdiagnosis, and overlap with conventional acute coronary syndromes [1,11]. Prospective emergency department data indicate that KS may account for up to 3–4% of patients presenting with allergic reactions [1,7]. Catheterization laboratory–based studies report a prevalence of approximately 0.002% among patients undergoing urgent coronary angiography for suspected acute myocardial infarction [10]. Higher incidence rates have been reported in Southern Europe and Mediterranean countries, possibly reflecting greater physician awareness, environmental exposures, dietary habits, and increased prevalence of allergic triggers [5]. Recent studies demonstrating elevated cardiac troponin levels in patients with anaphylaxis further support the concept that myocardial involvement during allergic reactions is more common than previously recognized [7].

KS is classified into three main variants [3,6]. Type I occurs in patients with angiographically normal or near-normal coronary arteries, where mast cell–mediated inflammatory mediator release induces coronary artery spasm, with or without myocardial necrosis [6]. Type II affects patients with pre-existing but clinically silent atherosclerotic disease, in whom allergic activation triggers coronary spasm, plaque erosion, or rupture, resulting in acute myocardial infarction [2,12]. Type III refers to allergic stent thrombosis and is characterized by the presence of eosinophils and mast cells within aspirated thrombi or adjacent coronary tissue, implicating hypersensitivity reactions to stent metals, polymers, or eluted drugs [1,5].

**Figure 1:**
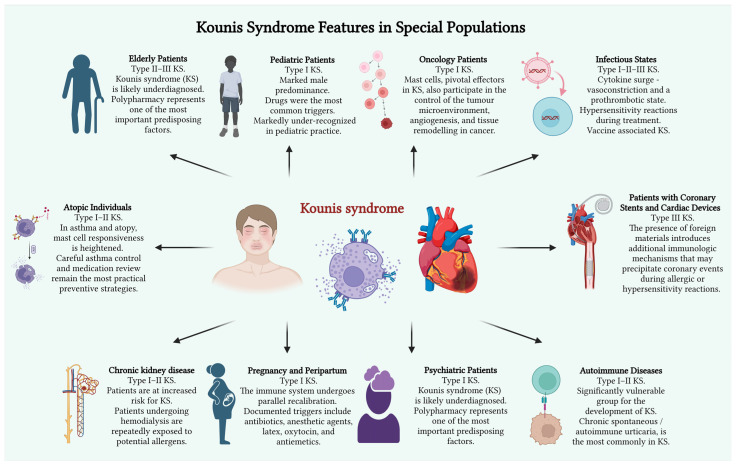
Kounis Syndrome Features in Special Populations. Created in BioRender. Ceasovschih, A. (2026) https://BioRender.com/4i0yfja (accessed on 20 April 2026).

This narrative review aims to summarize current evidence on KS across vulnerable host phenotypes, highlight diagnostic and therapeutic challenges in special populations, and propose a host-modified conceptual framework. These populations concentrate multiple risk modifiers, including polypharmacy, immune dysregulation, and altered vascular substrate, but are underrepresented in the existing literature (Figure 1).

Although this review is narrative rather than systematic, a combined strategy was employed to ensure comprehensive coverage of the literature and to enhance the transparency of the selection process. A literature search was conducted in the PubMed, Scopus, Web of Science, and Google Scholar databases for the period 1991–2025. The lower time limit was chosen based on the first description of the concept of “allergic angina,” which marked the beginning of research into KS [4]. The search was conducted using the following keywords and their combinations in English: “Kounis syndrome”, “allergic angina”, “allergic myocardial infarction”, “mast cells”, “coronary spasm”. The review included various types of publications: original studies, narrative and systematic reviews, and clinical case reports. Priority was given to studies published within the last 10 years. Earlier studies were included to describe historical aspects and fundamental pathophysiological concepts. Conference abstracts, preprints, and letters to the editor that did not contain original data were excluded from the analysis.

## 2. Elderly Patients

KS, defined as the concurrence of acute coronary syndromes with allergic, hypersensitivity, anaphylactic, or anaphylactoid reactions, has particular clinical relevance in elderly patients [6,7]. Aging is associated with a high burden of CV disease, immune system dysregulation, and extensive drug exposure, all of which create a permissive environment for the development of KS [13,14]. In older adults, KS is likely underdiagnosed, as allergic manifestations may be subtle, atypical, or masked by dominant CV symptoms.

Polypharmacy represents one of the most important predisposing factors for KS in the elderly [1,15]. Older patients are frequently exposed to multiple medications, including antibiotics, non-steroidal anti-inflammatory drugs, contrast media, antiplatelet agents, anticoagulants, antihypertensives, and proton pump inhibitors—many of which have been implicated as triggers of allergic reactions and KS [3,16]. Repeated exposure increases the risk of sensitization, while drug–drug interactions may amplify mast cell activation, platelet aggregation, and endothelial dysfunction, thereby facilitating coronary spasm, plaque destabilization, or stent thrombosis [1,6].

Aging is also associated with progressive endothelial dysfunction and increased plaque vulnerability [13,14]. Elderly patients typically present with advanced atherosclerosis characterized by lipid-rich plaques, thin fibrous caps, and increased inflammatory cell infiltration. Mast cell activation during allergic reactions promotes plaque erosion or rupture through the release of histamine, tryptase, chymase, leukotrienes, and platelet-activating factor [6,17]. These mediators induce coronary vasoconstriction, enhance platelet aggregation, activate matrix metalloproteinases, and increase tissue factor expression [14]. Consequently, Type II KS, which occurs in patients with pre-existing but quiescent coronary artery disease, represents the predominant variant in elderly individuals [3,5].

In addition, elderly patients frequently undergo coronary stent implantation, further predisposing them to Type III KS, characterized by allergic stent thrombosis [5,15]. Hypersensitivity reactions to stent components, including metals (nickel, chromium, cobalt), polymer coatings, and eluted drugs, may lead to persistent local inflammation, eosinophilic infiltration, and thrombus formation [15,16]. Age-related immune dysregulation, impaired endothelial repair, and prothrombotic states further increase susceptibility to this variant. Importantly, Type III KS often presents as acute or very late stent thrombosis with high morbidity and mortality, particularly in frail elderly patients [5].

Clinical recognition of KS in the elderly is challenging. Cutaneous or respiratory allergic symptoms may be absent, while hypotension, syncope, arrhythmias, acute heart failure, or cardiogenic shock may dominate the presentation [10,18]. Elevated cardiac biomarkers during allergic reactions should raise suspicion for KS, especially in patients with known coronary artery disease or prior coronary interventions [18]. Management is complex, as therapy must address both the allergic reaction and the acute coronary event, while avoiding medications that may worsen hypotension or coronary spasm [1].

In conclusion, KS in the elderly represents a clinically important but underrecognized condition driven by polypharmacy, plaque vulnerability, and prior coronary interventions. Type II and Type III variants predominate in this population, emphasizing the need for heightened clinical awareness, careful medication review, and individualized diagnostic and therapeutic strategies in older adults presenting with acute coronary syndromes in the context of allergic reactions [1,6,11]. That said, in older adults presenting with ACS and potential allergen exposure, routine review of recent medications and troponin elevation during anaphylaxis should prompt consideration of KS.

## 3. Pediatric Patients

Although initially described in adults, KS in pediatric cases have increasingly been reported, revealing distinct clinical and pathophysiological characteristics compared with older populations [3,8]. In children, Type I KS is the predominant variant, presenting as coronary vasospasm in the absence of structural coronary artery disease [5]. This form is considered part of the spectrum of myocardial infarction with non-obstructive coronary arteries (MINOCA) and reflects endothelial dysfunction and microvascular angina rather than plaque rupture or thrombosis [8]. Coronary angiography in pediatric cases is most often normal, reinforcing the vasospastic and reversible nature of myocardial ischemia in this age group [3]. Pediatric KS shows a strong association with IgE-mediated allergic reactions, with triggers differing significantly from adult populations. A systematic review of the literature identified pediatric cases with a median age in early adolescence and a marked male predominance [8]. Drugs were the most common triggers, particularly antibiotics, with amoxicillin/clavulanic acid accounting for the majority of cases [1,3]. Other reported pharmacological triggers included metronidazole, mesalamine, atropine, rocuronium, and inhalational anesthetics such as isoflurane [5,19]. Insect stings and bites—especially from bees, wasps, and spiders—represented the second most frequent trigger group, while food-related cases were less common but clinically significant [3,8].

Pathophysiologically, pediatric mast cell activation differs from adult patterns in mediator release and inflammatory signaling [2]. Children appear to exhibit a mediator profile dominated by histamine, leukotrienes, and platelet-activating factor, favoring intense but transient coronary vasoconstriction [6]. Importantly, elevated serum tryptase levels were documented in several pediatric cases, supporting mast cell activation as a central mechanism [3,8]. Unlike adults, chronic inflammatory pathways related to plaque vulnerability are absent, explaining the rarity of Type II and III variants in childhood.

Despite these mechanistic insights, KS remains markedly under-recognized in pediatric practice. Acute coronary syndromes are rare in children, leading to diagnostic bias whereby chest pain is often attributed to benign musculoskeletal, respiratory, or psychogenic causes [1]. In multiple reported cases, myocarditis was initially suspected before KS was recognized during follow-up [8,19]. Allergic symptoms may dominate the presentation, further delaying cardiac evaluation. Diagnostic assessment poses ethical and safety challenges. Coronary angiography, while useful to exclude congenital anomalies, carries procedural and radiation risks and is therefore not routinely performed in children [8]. As a result, diagnosis relies on the temporal relationship between allergen exposure and cardiac symptoms, electrocardiographic changes, transient elevation of cardiac biomarkers, and non-invasive imaging modalities such as echocardiography and cardiac magnetic resonance [18,20].

In conclusion, pediatric KS represents a distinct clinical entity dominated by Type I vasospastic mechanisms, strongly linked to allergic triggers—particularly antibiotics and insect stings. Increased awareness, careful allergy history, and judicious use of diagnostic tools are essential to avoid misdiagnosis and ensure optimal outcomes in this vulnerable population [6,8].

## 4. Patients with Coronary Stents and Cardiac Devices

KS arising in the context of coronary stents or cardiac implantable electronic devices constitutes a clinically important and mechanistically distinct subgroup within the broader spectrum of allergic acute coronary syndromes. The presence of intracoronary or intracardiac foreign material introduces additional immunologic triggers capable of precipitating or amplifying coronary events in the setting of allergic or hypersensitivity reactions. This interaction is increasingly relevant given the widespread use of drug-eluting stents and cardiac implantable electronic devices in contemporary CV practice [1,2].

In patients with coronary stents, KS most frequently manifests as Type III, which is characterized by acute stent thrombosis triggered by an allergic reaction. Hypersensitivity responses may develop against various stent components, including metallic alloys such as nickel, chromium, or cobalt, polymer coatings, or the antiproliferative drugs used for elution. Mast cell activation is central to this process, resulting in the release of histamine, leukotrienes, platelet-activating factor, and proinflammatory cytokines. These mediators promote intense platelet aggregation, coronary vasoconstriction, and thrombus formation, leading to abrupt vessel occlusion and acute myocardial infarction [2,21,22].

Clinically, patients with stent-related KS may present with sudden-onset chest pain, ST-segment elevation, cardiogenic shock, or malignant ventricular arrhythmias. Symptoms often occur in close temporal association with exposure to a known allergen, such as antibiotics, nonsteroidal anti-inflammatory drugs, iodinated contrast media, or insect stings. In some cases, systemic allergic manifestations may be mild or transient, making the diagnosis particularly challenging and increasing the risk of misclassification as primary stent failure or premature discontinuation of antiplatelet therapy [3,23].

Cardiac implantable electronic devices, including permanent pacemakers and implantable cardioverter-defibrillators, may also contribute to the development of KS. Hypersensitivity reactions to device components such as titanium casings, silicone insulation, polyurethane leads, or epoxy resins have been described. While localized reactions at the device pocket are more common, systemic immune activation may occur and indirectly provoke coronary artery spasm or thrombosis. In predisposed individuals, this immune response may serve as a trigger for KS, particularly when combined with other allergic stimuli or systemic inflammation [24,25,26].

Diagnosis of KS in this population remains complex. Coronary angiography may demonstrate acute stent thrombosis, diffuse coronary vasospasm, or angiographically normal coronary arteries, depending on the subtype of the syndrome. Device interrogation typically excludes primary electrical malfunction, while allergic symptoms may be subtle or overlooked. A thorough clinical history focused on recent allergen exposure, prior hypersensitivity reactions, and temporal symptom relationships is therefore essential for accurate diagnosis [11,27].

Management requires a careful balance between controlling the allergic reaction and treating the acute coronary event. Antihistamines and corticosteroids are fundamental for suppressing allergic inflammation, while nitrates and calcium channel blockers may alleviate coronary vasospasm. When stent thrombosis occurs, urgent revascularization and antiplatelet therapy remain the therapeutic cornerstone; clinicians should nonetheless bear in mind that antiplatelet agents themselves may, on rare occasions, serve as causative allergens. Beyond the acute phase, management should encompass allergologic workup, systematic avoidance of identified triggers, and—in patients with recurrent reactions—careful consideration of alternative stent platforms or device materials [28,29,30].

## 5. Patients with Chronic Kidney Disease and Dialysis

Patients with chronic kidney disease (CKD), particularly those receiving dialysis, are at increased risk for KS due to the coexistence of immune dysregulation, chronic systemic inflammation, endothelial dysfunction, and a high prevalence of CV disease. CKD is characterized by persistent oxidative stress, reduced nitric oxide bioavailability, and activation of proinflammatory and prothrombotic pathways, all of which facilitate exaggerated coronary responses following allergic or hypersensitivity stimuli. These mechanisms create a vulnerable CV substrate in which allergic reactions may readily precipitate acute coronary events [31,32,33].

Uremia is associated with profound alterations in both innate and adaptive immune responses, including impaired T-lymphocyte function, increased mast cell activation, and elevated circulating inflammatory cytokines. Mast cells play a central role in the pathophysiology of KS through the release of histamine, leukotrienes, prostaglandins, platelet-activating factor, and cytokines. These mediators can induce coronary vasospasm, endothelial injury, plaque destabilization, and thrombosis, thereby linking allergic inflammation directly to myocardial ischemia and infarction [4,11,34].

Patients undergoing hemodialysis are repeatedly exposed to a wide range of potential allergens, including dialysis membranes, tubing materials, ethylene oxide sterilizing agents, anticoagulants such as heparin, antibiotics, intravenous medications, and iodinated contrast media. Hypersensitivity reactions during dialysis sessions are well recognized and may range from mild cutaneous symptoms to severe anaphylaxis with CV collapse. In susceptible individuals, such reactions may trigger mast cell degranulation and precipitate KS, leading to acute myocardial ischemia in temporal association with allergic manifestations [35,36].

Clinical recognition of KS in patients with CKD is particularly challenging. Typical ischemic chest pain may be absent or atypical due to autonomic dysfunction, diabetic neuropathy, or altered pain perception. Baseline electrocardiographic abnormalities, including left ventricular hypertrophy and conduction disturbances, are common and may obscure acute ischemic changes. Furthermore, interpretation of cardiac biomarkers is complicated by chronically elevated troponin levels frequently observed in advanced renal disease, resulting in misdiagnosis or underrecognition of allergic acute coronary events [27,37].

Coronary angiographic findings in CKD patients with suspected KS are heterogeneous and may include angiographically normal coronary arteries with reversible vasospasm, diffuse atherosclerotic disease, or acute thrombotic occlusions. Extensive vascular calcification and impaired vasomotor function, hallmarks of advanced CKD, may amplify the deleterious effects of allergic inflammatory mediators on the coronary circulation. Additionally, exposure to iodinated contrast during diagnostic or interventional procedures represents a significant trigger for hypersensitivity reactions and may directly precipitate KS [1,23].

Management of KS in patients with CKD and those on dialysis requires an individualized and carefully balanced therapeutic approach. Corticosteroids and antihistamines are generally safe and effective for controlling allergic inflammation, while coronary vasospasm may be treated with nitrates and calcium channel blockers. Standard acute coronary syndrome therapies must be adjusted according to renal function and bleeding risk. Although epinephrine remains essential for the treatment of anaphylaxis, it should be administered cautiously due to its potential to exacerbate myocardial ischemia. Preventive strategies, including identification of prior hypersensitivity reactions, use of biocompatible dialysis materials, and avoidance of known allergens, are essential to reduce recurrence [28,29,38].

## 6. Pregnancy and the Peripartum Period

Pregnancy alters CV physiology in ways that are both adaptive and potentially destabilizing under stress. Plasma volume nearly doubles, cardiac output rises substantially, and systemic vascular resistance falls. Coronary circulation becomes more reactive to neurohumoral and inflammatory stimuli [39,40]. In most women, these changes are well tolerated. However, when an intense allergic reaction occurs, the same physiologic adjustments can amplify ischemic vulnerability.

The immune system undergoes parallel recalibration. A shift toward T helper 2 predominance supports maternal–fetal tolerance but also enhances humoral and immunoglobulin E mediated responses [41,42]. Mast cells in this setting may respond more vigorously to allergens. In KS, mast cell degranulation releases histamine, leukotrienes, platelet-activating factor, and proinflammatory cytokines, leading to coronary vasospasm, endothelial dysfunction, and in some cases plaque destabilization [2,43].

It is important to distinguish mechanistic subtypes. In patients without preexisting coronary disease, allergic mediator release typically induces intense vasospasm consistent with Type I KS [43,44]. In women with underlying atherosclerosis, inflammatory activation may precipitate plaque erosion or rupture, corresponding to Type II. Pregnancy itself is not strongly associated with obstructive coronary disease, but spontaneous coronary artery dissection (SCAD) and endothelial instability are increasingly recognized in the peripartum period [45,46]. SCAD warrants special attention as a non-atherosclerotic cause of myocardial infarction, particularly during pregnancy and the postpartum period [47,48]. The etiology of SCAD remains incompletely understood, but proposed mechanisms include hemodynamic stress, hormonal fluctuations, and inflammation, including autoimmune inflammation [49].

Cases of coronary artery dissection caused by allergic or anaphylactic reactions are extremely rare. Nevertheless, some cases of dissection are associated with eosinophilic coronary periarteritis (ECPA), in the pathogenesis of which mast cells are believed to play a role [50]. In a recently described clinical case, a middle-aged woman experienced two episodes of acute coronary syndrome—one triggered by an allergic reaction, the other by a non-allergic mechanism—both of which led to coronary artery dissection [51]. This case suggests that in predisposed individuals, inflammatory activation (including mast cell degranulation) may contribute to the development of SCAD, potentially KS with SCAD. However, direct evidence of this link is currently insufficient, and the issue requires further investigation. These overlapping substrates may complicate presentation and management.

The uteroplacental circulation introduces a second hemodynamic system dependent on maternal stability. During anaphylaxis, systemic hypotension, abrupt shifts in vascular tone, and microvascular dysfunction can reduce uterine perfusion pressure. Placental blood flow lacks strong autoregulatory capacity, making it sensitive to maternal hemodynamic fluctuation. Inflammatory mediators may further impair placental endothelial nitric oxide signaling and promote microthrombotic phenomena [52]. Even transient reductions in perfusion may result in fetal hypoxia.

Clinical evidence remains limited largely to case reports and small series. Documented triggers include antibiotics, anesthetic agents, latex, oxytocin, and antiemetics such as ondansetron [53,54,55]. Broader analyses confirm that perioperative drug exposure is among the most common precipitants of allergic acute coronary syndromes [1,56]. Prospective pregnancy-specific registries are lacking.

Management requires careful prioritization. Epinephrine remains essential in life-threatening anaphylaxis. Although theoretical concern exists regarding coronary spasm and uteroplacental vasoconstriction, delayed administration carries greater maternal risk. Stabilization of maternal circulation is the primary determinant of fetal survival. Adjunctive therapies, including cautious vasodilator use and invasive monitoring, when necessary, must be individualized [44,57].

Pregnancy associated KS therefore represents a convergence of immune amplification, vascular reactivity, and dual circulatory dependence. Recognition depends less on rarity and more on clinical suspicion when allergic and ischemic symptoms coexist.

## 7. Atopic Individuals and Asthmatics

Current evidence on the association between KS and asthma is largely hypothesis-generating and based on mechanistic parallels rather than robust epidemiologic data. Chronic atopic disease is characterized by sustained type 2 immune activation, elevated immunoglobulin E, and persistent mast cell priming. While traditionally viewed as organ specific, this inflammatory state extends beyond the airways. Coronary vessels are not immunologically insulated. Mast cells reside within the adventitia and shoulder regions of atherosclerotic plaques, where mediator release can influence vascular tone and plaque stability [2,58].

In asthma and atopy, mast cell responsiveness is heightened. Activation results in release of histamine, tryptase, leukotrienes, platelet-activating factor, and cytokines capable of inducing coronary smooth muscle contraction, endothelial dysfunction, and platelet aggregation [1,59]. Leukotrienes, central to bronchoconstriction, also exert potent vasoconstrictive effects within coronary microcirculation [45,60,61,62]. This shared mediator biology explains why severe allergic reactions may present with simultaneous respiratory compromise and myocardial ischemia.

Observational reports describe recurrent Kounis episodes in patients with poorly controlled asthma or multiple drug hypersensitivities [1,53]. However, most evidence derives from retrospective case-based literature. Quantitative recurrence risk and long-term CV outcomes remain insufficiently characterized.

Biologic therapies targeting type 2 inflammation have transformed severe asthma care. Omalizumab reduces circulating immunoglobulin E, whereas dupilumab inhibits interleukin 4 and interleukin 13 signaling pathways. Randomized trials demonstrate reduced exacerbations and improved symptom control [63,64]. Whether these agents confer direct CV benefit remains uncertain. Recent population analyses suggest an association between biologic therapy and reduced CV events. Biologic therapies for patients with severe asthma were associated with a significantly lower risk of all-cause mortality and specific CV diseases compared with non-biologic users [65,66,67], but residual confounding cannot be excluded. Furthermore, rare hypersensitivity reactions to biologics themselves have been reported.

Thus, while improved inflammatory control may plausibly reduce susceptibility to allergic coronary syndromes, definitive prospective CV outcome data are lacking. At present, careful asthma control and medication review remain the most practical preventive strategies.

## 8. COVID-19 and Post-Infectious States

KS cases in Coronavirus disease 2019 (COVID-19) remain rare and predominantly case-based. In terms of pathophysiology, COVID-19 offers a striking illustration of how immune activation, endothelial biology, and coronary physiology converge under systemic stress. Severe acute respiratory syndrome coronavirus 2 (SARS-CoV-2) gains cellular entry via angiotensin-converting enzyme 2 receptors expressed on endothelial surfaces throughout both the coronary and systemic vasculature. Beyond serving as a viral entry point, this interaction disrupts endothelial homeostasis, diminishes nitric oxide bioavailability, enhances oxidative stress, and activates proinflammatory signaling cascade [68,69,70,71]. The net effect is a shift away from physiologic vasodilation and antithrombotic balance toward vasoconstriction and a prothrombotic state. Autopsy studies consistently demonstrate diffuse microvascular injury, endothelial swelling, and intramyocardial thrombosis, frequently in the absence of obstructive epicardial coronary artery disease [72], highlighting the inflammatory and microvascular nature of COVID-related cardiac injury [73,74].

The cytokine response elicited by acute infection, characterized by marked elevations in interleukin-6 and tumor necrosis factor, amplifies leukocyte adhesion, upregulates tissue factor expression, and promotes platelet activation [75,76]. This inflammatory milieu impairs coronary vasodilatory reserve and may unmask or destabilize previously silent endothelial dysfunction. Importantly, vascular abnormalities may persist beyond viral clearance. Sustained endothelial dysfunction and elevated inflammatory or prothrombotic biomarkers have been documented during convalescence, suggesting prolonged vascular remodeling and ongoing CV susceptibility [77,78].

The spectrum of myocardial injury in COVID-19 is heterogeneous. Cardiac magnetic resonance imaging has identified immune mediated myocarditis characterized by myocardial edema and inflammatory infiltration in some patients, whereas others demonstrate patterns consistent with microvascular ischemia or myocardial infarction with non-obstructive coronary arteries [72]. These overlapping phenotypes reinforce the concept that endothelial dysfunction, immune dysregulation, and coronary vasomotor instability may coexist within the same individual.

Within this proinflammatory and endothelial vulnerable environment, rare presentations consistent with KS have been described during or shortly after SARS-CoV-2 infection. Case reports document episodes of urticaria accompanied by coronary vasospasm and myocardial ischemia, suggesting mast cell activation superimposed on systemic inflammation as a potential trigger. Additionally, hypersensitivity reactions during treatment, such as drug-induced allergic responses in patients hospitalized with COVID-19 pneumonia, have been temporally associated with acute coronary syndromes characteristic of KS [79,80]. Hypersensitivity reactions to therapeutic monoclonal antibodies have also been linked to allergic coronary events in infected individuals [81]. Although largely limited to case-based evidence, the biological plausibility is compelling given the convergence of cytokine excess, endothelial dysfunction, platelet activation, and immune priming.

Vaccine-associated cardiac inflammation has also been systematically studied. Myocarditis following mRNA vaccination has been documented, predominantly in young males, though large population-based analyses consistently demonstrate that SARS-CoV-2 infection itself carries a substantially higher risk of myocarditis than vaccination [82,83]. Rare cases of KS temporally associated with COVID-19 vaccination have also been described, with proposed mechanisms involving hypersensitivity responses and mast cell activation leading to coronary vasospasm or plaque destabilization [84]. These events remain uncommon, and contextualizing relative risk is essential to balanced CV risk assessment.

SARS-CoV-2 infection creates a coronary environment defined by endothelial fragility, amplified inflammatory signaling, oxidative stress, and altered vasomotor regulation. In this context, allergic coronary syndromes such as KS remain uncommon, yet biologically plausible. Understanding how immune activation intersects with coronary physiology in this setting may enhance post-infectious CV assessment and provide broader insight into inflammation-driven coronary disorders.

## 9. Autoimmune and Inflammatory Diseases Patients

Patients with autoimmune and chronic inflammatory diseases represent a significantly vulnerable group for the development of KS, due to shared immunological mechanisms, increased exposure to triggering factors, and considerable diagnostic overlap with other inflammatory cardiac conditions. In these patients, allergic acute coronary syndromes may be insufficiently identified or miscategorized, leading to delays in appropriate treatment [44].

Chronic spontaneous urticaria, in particular its autoimmune form, chronic autoimmune urticaria, is the most commonly associated autoimmune disorder to KS (allergic acute coronary syndrome), as evidenced by case reports and literature reviews that highlight its role as both a trigger and a comorbidity in recurrent and acute occurrences of KS. A recent case report further illustrates this association, describing Type I KS in a patient with chronic spontaneous urticaria who developed MINOCA due to coronary artery spasm, with the diagnosis of urticaria being established only during hospitalization [85]. The pathophysiological connection encompasses prolonged mast cell activation, frequently driven by autoantibodies targeting the high-affinity IgE receptor, which may trigger coronary events during urticarial episodes [86,87]. Other autoimmune disorders have been sporadically recorded in the context of KS; nonetheless, the medical literature predominantly emphasizes chronic spontaneous urticaria as the prototypical autoimmune illness in this setting. A case report exists of KS in a patient with Samter’s triad (aspirin-exacerbated respiratory disease), characterized by chronic rhinosinusitis with nasal polyps and asthma; nevertheless, this syndrome is not traditionally classified as an autoimmune disorder [87,88].

No substantial evidence presently corroborates a frequent correlation between KS and other systemic autoimmune disorders, such systemic lupus erythematosus, rheumatoid arthritis, or vasculitides, as per the studied medical literature. On the other hand, common inflammatory pathways connect autoimmune diseases with KS. Elevated cytokines such as IL-6 and TNF-α in chronic systemic inflammation result in endothelial dysfunction, oxidative stress, and a prothrombotic state. Through cytokine secretion, antigen presentation, and T-lymphocyte interaction, mast cells play a role in autoimmune inflammation and KS. Within an already inflamed vascular environment, sustained immunological activation lowers the threshold for acute mast cell–mediated coronary events, thereby supporting KS as a “second-hit” phenomenon [55,89,90,91]. Furthermore, in autoimmune disorders, autoreactive IgE antibodies can sensitize mast cells, leading to degranulation upon antigen recognition and the activation of FcεRI-mediated signaling, involving downstream PLCγ, PI3K–AKT, and MAPK pathways. This is analogous to KS, in which allergen-specific IgE cross-links mast cells, resulting in the release of histamine, tryptase, leukotrienes, and cytokines that provoke coronary vasospasm and platelet activation. The common IgE–mast cell axis underscores interconnected immune–metabolic pathways, associating chronic autoimmunity with increased vulnerability to allergic acute coronary incidents [92]. Additionally, mast cell-derived chymase stimulates matrix metalloproteinase-9 (MMP-9) in autoimmune disorders, facilitating vascular remodeling and tissue damage. In KS, chymase-mediated MMP-9 plays a role in destabilizing coronary plaques and inducing vasospasm, hence connecting mast cell activation to acute coronary incidents [93]. This indicates that individuals with autoimmune diseases may have a heightened susceptibility to KS, as persistent systemic inflammation predisposes the CV system to acute mast cell–mediated episodes. Therefore, KS may serve as a clinical manifestation indirectly associated with underlying autoimmune pathology.

## 10. Oncological Patients

There is strong overlap and interaction between the pathophysiological pathways of cancer and KS. Mast cells, pivotal effectors in KS, also participate in the control of the tumor microenvironment, angiogenesis, and tissue remodeling in cancer. Oxidative stress and endothelial dysfunction, prevalent in both cancer and CV disease, establish a further connection between these illnesses. Cancer treatments, especially those that provoke hypersensitivity reactions may elicit KS in predisposed patients, demonstrating a direct clinical connection between oncology and allergic coronary syndromes [6,94,95]. In oncology patients, prevalent risk factors for KS encompass exposure to pharmacological substances that induce hypersensitivity reactions, including chemotherapeutic medicines (e.g., epirubicin, cisplatin, cyclophosphamide) and monoclonal antibodies utilized in cancer treatment [96,97,98]. Giving iodinated contrast substances for diagnostic imaging is another common cause in this group of people [55,96]. Pre-existing coronary artery disease, hypertension, diabetes mellitus, dyslipidemia, and smoking are all CV risk factors that make KS more likely [44,99]. Since the primary pathophysiological mechanism underlying KS is allergic in nature, most cases reported in oncology patients are attributable to medications used in the course of cancer treatment. The agents most commonly implicated are those whose adverse effect profiles include allergy, hypersensitivity, or direct vascular injury. The following drug classes and agents are of particular relevance:

- Monoclonal antibodies (e.g., rituximab, bevacizumab, ramucirumab, trastuzumab, pertuzumab, alemtuzumab, aflibercept). These drugs may induce systemic hypersensitivity reactions, including cutaneous erythema, pruritus, chills, and chest discomfort, which can precede cardiac events such as coronary vasospasm, angina, myocardial infarction, and arrhythmias. IgE-mediated reactions have been recorded, corroborating the mechanism of KS in this setting [97,100].
- Antimetabolites, particularly 5-fluorouracil and capecitabine, are recognized for their capacity to provoke coronary vasospasm, potentially resulting in angina or myocardial infarction, even in the absence of preexisting coronary artery disease. Symptoms typically manifest swiftly following medication intake and may be associated with arrhythmias and alterations in the ECG [100,101].
- Platinum agents: Cisplatin is linked to acute coronary thrombosis and vasospasm, presumably resulting from endothelial damage, platelet activation, and aggregation. These effects can trigger acute coronary syndromes during hypersensitivity reactions [96,100].
- Microtubule inhibitors (paclitaxel, docetaxel, vincristine, vinblastine) have been associated with case reports of myocardial ischemia and infarction, frequently due to coronary vasospasm [98].
- Tyrosine kinase inhibitors (nilotinib, ponatinib, sunitinib, sorafenib, pazopanib) are correlated with angina, myocardial infarction, and the advancement of coronary artery disease, with certain incidents associated with hypersensitivity or vasospastic processes [100,101].
- Immune checkpoint inhibitors (ipilimumab, nivolumab, pembrolizumab): Although myocarditis and pericarditis are more prevalent, these agents have also been linked to vasculitis and atherosclerotic events, perhaps intersecting with the pathophysiology of KS in the realm of immune-mediated hypersensitivity [102].

In summary, the possible adverse effects of oncology drugs that may induce KS encompass systemic allergic reactions, coronary vasospasm, angina, myocardial infarction, arrhythmias, and acute coronary thrombosis. Furthermore, the common pathophysiological mechanisms establish a pathophysiological continuum between cancer development/progression and KS, featuring overlapping mediators and cellular pathways that may interact and enhance one another in oncology patients [96,98,99,100,101,102].

## 11. Psychiatric and Neurodevelopmental Populations

The correlation between psychiatric and neurodevelopmental issues and KS (allergic acute coronary syndrome) is not explicitly examined in the existing medical literature, including recent systematic reviews and epidemiological research. No research has explicitly measured the prevalence of KS in populations with psychiatric or neurodevelopmental disorders, nor have these illnesses been recognized as independent risk factors for KS [44,50].

Although psychiatric and neurodevelopmental disorders are associated with altered autonomic function, heightened systemic inflammation, and, in some cases, increased rates of atopic disease, no direct evidence currently establishes a causal link between these mechanisms and an elevated risk of KS. No distinct pathophysiological pathway directly linking psychiatric or neurodevelopmental disorders to the immunologic cascade underlying KS has yet been established. That said, autonomic dysfunction offers a plausible mechanistic bridge. Stress, anxiety, and mood disorders are characterized by sympathetic overactivity and parasympathetic withdrawal, a pattern that facilitates mast cell degranulation and vascular instability. This same autonomic imbalance may predispose susceptible individuals to coronary vasospasm and arrhythmias during allergic or inflammatory insults. Thus, psychological stress, immune activation, and vascular reactivity form a tightly interwoven triad [103,104].

In mental health conditions including major depression, bipolar disorder, schizophrenia, and autism spectrum syndromes, increasing evidence suggests that persistent low-grade neuroinflammation is a more significant factor than mere neurotransmitter imbalance. Mast cells, stimulated by IgE as well as stress-induced corticotropin-releasing hormone, neuropeptides such as substance P, and complement fragments, can compromise the blood–brain barrier and enhance inflammatory signaling. The released mediators stimulate microglia, the brain’s resident immune cells, establishing a self-sustaining inflammatory loop. Significantly, numerous cytokines that are high in acute KS, such as IL-1β, IL-6, and TNF-α, are significantly correlated with the severity of depressive symptoms, cognitive impairment, and resistance to treatment [105].

No evidence exists to suggest that psychiatric or neurodevelopmental comorbidities modify the clinical presentation, diagnostic criteria, or therapeutic protocols for KS. Nonetheless, considering that numerous psychotropic medications and agents utilized in neurodevelopmental disorders may be allergenic or interact with CV and immune systems, meticulous selection of medications and monitoring for allergic reactions may be advisable, although this is not explicitly addressed in the literature regarding KS. The existing medical literature does not demonstrate a direct correlation between psychiatric or neurodevelopmental diseases and KS regarding prevalence, etiology, or therapy. Clinical concerns for drug selection or diagnostic vigilance in these groups must be derived from general principles rather than data unique to KS [5,10,106].

On the other hand, there is a growing body of evidence regarding the role of mast cells in central nervous system function and neurological disorders resulting from disruptions in the normal balance of mast cell activity. The most striking clinical example of such a bidirectional relationship is systemic mastocytosis and mast cell activation syndrome (MCAS), in which mast cell hyperreactivity affects many organs, including the CV system [107], such as KS [108,109]. Systemic mastocytosis and MCAS are also associated with various neurological and psychiatric disorders, including headache, dysautonomia, depression, generalized anxiety disorder, and many others [110,111,112]. Thus, MCAS represents a unique example demonstrating that mast cell activation can affect the central nervous system and coronary circulation. In this regard, new research on the role of mast cells in the CNS and their connection to other organs is a promising area of study.

## 12. Unifying Framework: Host-Modified Kounis Syndrome

**Figure 2:**
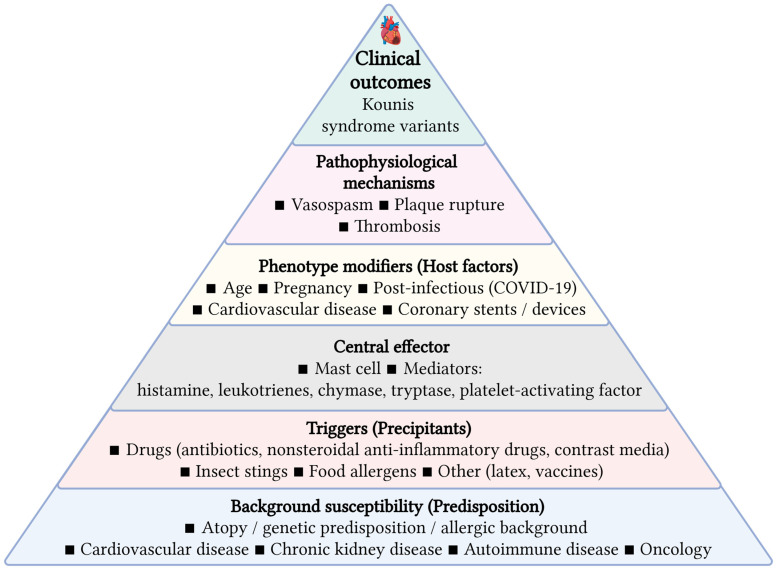
Pyramid of Risk Factors and Pathophysiological Mechanisms in Kounis Syndrome. Created in BioRender. Ceasovschih, A. (2026) https://BioRender.com/0t07ir3 (accessed on 20 April 2026).

Host variables, such as age, genetic predisposition, comorbidities, immunological status, and pre-existing allergy or inflammatory tendencies, significantly affect the manifestation, pathogenesis, and clinical progression of KS (Figure 2). Age is a crucial factor; KS is predominantly documented in middle-aged and older individuals, with average ages in case series varying from 54 to 66 years. Older patients exhibit a greater prevalence of underlying coronary artery disease, rendering them susceptible to more severe manifestations (e.g., ST-elevation myocardial infarction, arrhythmias, and elevated mortality) and to type II or III KS, wherein allergic reactions trigger plaque rupture or stent thrombosis. Young patients, even those lacking conventional CV risk factors, can nonetheless develop KS, frequently presenting as coronary vasospasm (type I) and may exhibit atypical symptoms, perhaps without chest discomfort [10,106,107].

The genetic background and atopic history are significant. A significant percentage of individuals with KS had a personal or familial history of atopy, asthma, or allergy conditions, indicating a hereditary susceptibility to increased mast cell reactivity and IgE-mediated reactions. This inherent allergic predisposition heightens vulnerability to KS and may diminish the threshold for cardiac involvement during systemic allergic reactions [44].

Comorbidities including hypertension, diabetes mellitus, dyslipidemia, and pre-existing coronary artery disease correlate with increased cardiac damage severity and poorer outcomes in KS. These conditions lead to endothelial dysfunction and increase the susceptibility of atherosclerotic plaques, heightening the risk of acute coronary events when confronted with systemic inflammatory mediators generated during allergic reactions. Patients with these comorbidities are more predisposed to myocardial infarction than to isolated vasospasm and exhibit elevated rates of sequelae, including arrhythmias, stroke, and increased in-hospital mortality [44,50,86,87,88,109].

A baseline allergic predisposition, encompassing previous incidents of anaphylaxis or severe medication allergies, constitutes a significant risk factor for KS. Individuals with a history of severe allergic reactions exhibit an increased propensity for coronary involvement during repeated exposures, and the clinical trajectory may be more acute, characterized by fast progression to hemodynamic instability or cardiac arrest. All above mentioned variables dictate whether the illness presents as solitary vasospasm or acute myocardial infarction, influence the extent of heart damage, and impact prognosis [6,89,90,92,96].

Based on the proposed framework, patients can be pragmatically stratified into low-, intermediate-, and high-risk profiles according to allergic burden and underlying coronary substrate. Low-risk presentations typically involve isolated vasospasm in structurally normal coronaries (Type I KS), whereas intermediate-risk cases reflect moderate allergic activation in patients with coronary artery disease (Type II KS). High-risk profiles are characterized by severe hypersensitivity reactions in the presence of stents, devices, or advanced vascular pathology, corresponding to Type III KS and increased risk of thrombosis and adverse outcomes.

This approach links immunologic activation with coronary vulnerability and may support early recognition and risk-adapted clinical management in acute settings [8].

**Figure 3:**
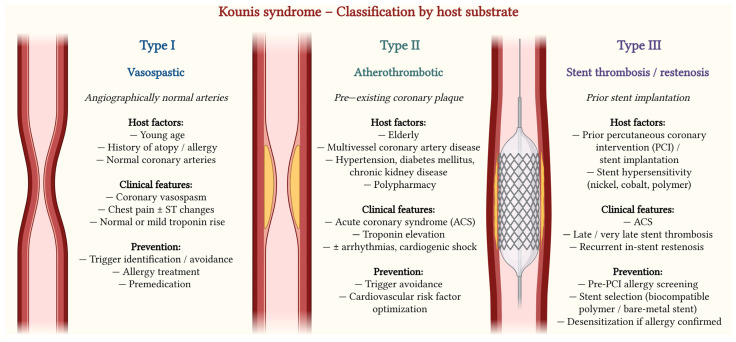
Kounis syndrome—classification by host factors. Created in BioRender. Ceasovschih, A. (2026) https://BioRender.com/tyglx56 (accessed on 20 April 2026).

This host-modified framework supports the development of pragmatic risk-stratification approaches integrating allergic history, mast cell–driven disease burden, and coronary substrate in emergency settings. It also implies that prevention should be phenotype-tailored: Type I–dominant presentations may benefit most from trigger avoidance and optimized allergy control, whereas Type II/III phenotypes warrant intensified CV risk optimization, careful antithrombotic decision-making, and thoughtful device/stent selection (Figure 3).

## 13. Conclusions

KS represents an underrecognized entity at the interface of allergy and acute coronary disease, whose expression is strongly shaped by host factors. Age, comorbidity burden, immune phenotype, and underlying vascular substrate collectively determine presentation and outcomes. Elderly and chronically ill patients more often exhibit plaque destabilization or stent-related events, whereas pediatric cases are usually vasospastic with normal coronaries. Pregnancy, atopy, autoimmune disease, chronic kidney disease, cardio-oncology exposures, and post-infectious states further modulate risk and can accelerate clinical deterioration. Accordingly, KS should be incorporated into ACS differentials in emergency and perioperative settings, particularly in elderly, pediatric, CKD, pregnant, cardio-oncology, and highly atopic patients. Greater interdisciplinary awareness and routine integration of immunologic and CV assessment are essential to enable timely recognition and phenotype-directed management. Prospective registries stratified by host phenotype and coronary substrate are needed to validate this host-modified framework and to guide tailored diagnostic and therapeutic algorithms, improving risk stratification, prevention, and personalized care.

## Data Availability

The original contributions presented in this study are included in the article/supplementary material. Further inquiries can be directed to the corresponding author(s).
